# Prenatal Nutritional Factors and Neurodevelopmental Disorders: A Narrative Review

**DOI:** 10.3390/life14091084

**Published:** 2024-08-29

**Authors:** Federica Cernigliaro, Andrea Santangelo, Rosaria Nardello, Salvatore Lo Cascio, Sofia D’Agostino, Edvige Correnti, Francesca Marchese, Renata Pitino, Silvia Valdese, Carmelo Rizzo, Vincenzo Raieli, Giuseppe Santangelo

**Affiliations:** 1 Department of Health Promotion, Mother and Child Care, Internal Medicine and Medical Specialities “G. D’Alessandro”, University of Palermo, 90127 Palermo, Italy; fede.cernigliaro@gmail.com (F.C.); rosaria.nardello@unipa.it (R.N.); salvatorelocascio.93@gmail.com (S.L.C.); sofiadagostino94@gmail.com (S.D.); 2 Pediatrics Department, AOUP Santa Chiara Hospital, 56126 Pisa, Italy; androsantangelo@gmail.com; 3 Department of Neurosciences, Rehabilitation, Ophthalmology, Genetics, Maternal and Child Health, University of Genoa, 16126 Genoa, Italy; 4 Child Neuropsychiatry Department, ISMEP—ARNAS Civico–Di Cristina Benfratelli, Di Cristina Pediatric Hospital, 90134 Palermo, Italy; edvigecorrenti@gmail.com (E.C.); renapiti@gmail.com (R.P.); dvaldese@hotmail.it (S.V.); giuseppe.santangelo@arnascivico.it (G.S.); 5 UOC NPIA—ASP Palermo, 90142 Palermo, Italy; dr.marchesefrancesca@gmail.com; 6 A.I.Nu.C—International Academy of Clinical Nutrition, 00166 Rome, Italy; info@carmelorizzo.it

**Keywords:** neurodevelopmental disorders, maternal nutrition, autism spectrum disorder, prenatal nutritional factors, environmental factors

## Abstract

According to the DSM-5, neurodevelopmental disorders represent a group of heterogeneous conditions, with onset during the developmental period, characterized by an alteration of communication and social skills, learning, adaptive behavior, executive functions, and psychomotor skills. These deficits determine an impairment of personal, social, scholastic, or occupational functioning. Neurodevelopmental disorders are characterized by an increased incidence and a multifactorial etiology, including genetic and environmental components. Data largely explain the role of genetic and environmental factors, also through epigenetic modifications such as DNA methylation and miRNA. Despite genetic factors, nutritional factors also play a significant role in the pathophysiology of these disorders, both in the prenatal and postnatal period, underscoring that the control of modifiable factors could decrease the incidence of neurodevelopmental disorders. The preventive role of nutrition is widely studied as regards many chronic diseases, such as diabetes, hypertension, and cancer, but actually we also know the effects of nutrition on embryonic brain development and the influence of prenatal and preconceptional nutrition in predisposition to various pathologies. These factors are not limited only to a correct caloric intake and a good BMI, but rather to an adequate and balanced intake of macro and micronutrients, the type of diet, and other elements such as exposure to heavy metals. This review represents an analysis of the literature as regards the physiopathological mechanisms by which food influences our state of health, especially in the age of development (from birth to adolescence), through prenatal and preconceptional changes, underlying how controlling these nutritional factors should improve mothers’ nutritional state to significantly reduce the risk of neurodevelopmental disorders in offspring. We searched key words such as “maternal nutrition and neurodevelopmental disorders” on Pubmed and Google Scholar, selecting the main reviews and excluding individual cases. Therefore, nutrigenetics and nutrigenomics teach us the importance of personalized nutrition for good health. So future perspectives may include well-established reference values in order to determine the correct nutritional intake of mothers through food and integration.

## 1. Introduction

A correct lifestyle based on healthy nutrition and physical activity is essential for maintaining good health. 

The preventive role of nutrition is widely studied concerning many chronic diseases, such as diabetes, hypertension, and cancer. Today, we also understand the effects of nutrition on fetal brain development and the influence of prenatal and preconceptional nutrition on the predisposition to various pathologies. These factors extend beyond correct caloric intake and a good BMI, emphasizing the importance of a balanced intake of macro and micronutrients, the type of diet, and other elements. Unfortunately, we must also consider possible exposure to harmful environmental factors that can be ingested through diet, such as heavy metals.

This review investigates the influence of prenatal nutritional factors on neurodevelopmental disorders, ever-increasing pathologies characterized by a multifactorial etiology involving an interplay of both genetic and environmental components.

Therefore, this study represents an analysis of the literature regarding the physiopathological mechanisms by which food influences our state of health, especially during the age of development, through prenatal and preconceptional changes.

## 2. Preventive Nutrition

The importance of nutrition has been recognized since the time of Hippocrates (460–377 BC), who stated: “If we were able to provide everyone with the right amount of nutrition and physical exercise, neither in deficiency nor in excess, we would have found the road to health”, but also “Let food be your medicine and let your medicine be food”. In 1848 Ludwig Feuerbach, a German philosopher, stated, “Man is what he eats”.

These statements, like many others throughout history, highlight that correct nutrition is crucial for psycho–physical health and for the prevention of various pathologies; in fact, eating habits, such as high consumption of fats and sugars, street food, alcohol abuse, and reduced intake of fruit and vegetables, constitute risk factors for many chronic diseases [1]. Therefore, an appropriate nutritional pattern is recommended as a protective factor against heart disease, cancer, diabetes, and various other pathologies, which represent the primary cause of morbidity and mortality worldwide. 

Recently, much attention has been paid to the role played by diet in human reproduction [1], underlying the impact of the prenatal environment, or even preconceptional one, on long-term health in offspring.

Furthermore, diet constitutes an important variable in the development and modification of microbiota. For example, ketogenic diet (KD) influences gut microbial composition [2], which plays an important role in different diseases, including neurodevelopmental disorders. KD can lead to epigenetic mechanisms and gut microbiota changes, suggesting its potential therapeutic role in pathologies such as refractory epilepsy and obesity [2].

Nutrition is a modulating factor capable of interfering with the genome and the epigenome, influencing human health and fertility and, therefore, the quality of life [1]. For example, specific genetic variants can influence the response to the components of diet and nutritional needs, while the diet can modulate gene expression.

Nutrigenetics can be defined as the field of nutritional genomics studying the role of specific genetic variants, including single nucleotide polymorphisms (SNP), the modulation of response to dietary components, and the implications of this interaction, including the influence on health status and susceptibility to nutrition-related diseases.

Epigenetics focuses on the molecular processes modulating gene expression without changing the DNA sequence, such as DNA methylation, modification of histones, and regulation of microRNAs (miRNAs). Nutrition can lead to epigenetic regulation through DNA methylation [1]; in fact, nutritional experiences in the first years of life or prenatal and postnatal exposure to different elements through maternal nutrition can induce persistent metabolic and physiological changes, derived from altered epigenetic profiles, which lead to different susceptibility to various chronic diseases in later stages of life.

Maternal nutrition is an important cause of epigenetic modifications in the embryo [1]: micronutrients influence the methylation of DNA, while alterations in lipids and dietary sugars influence mitochondrial activity. 

Obesity can disrupt female fertility by affecting processes such as sex hormone secretion, oocyte differentiation and maturation, and endometrial implantation. Bariatric surgery has been shown to improve fertility, reduce pregnancy complications, and enhance fetal health. This connection is partly mediated by the gut microbiome, where imbalances in obese individuals lead to increased levels of lipopolysaccharides (LPS), endotoxemia, and proinflammatory cytokines like IL-6 and IL-1β. These factors can decrease oocyte quality and disrupt reproductive functions through inflammation [3]. Some studies demonstrated that oocyte quality, mitochondrial function, and fertility in animal models can be restored by caloric restriction or omega-3 supplementation.

Additionally, the microbiota in the digestive tract shares similarities with the female reproductive tract. Alterations in this microbiome continuum, including before pregnancy, can have significant implications for maternal and fetal health. For instance, women who had a high BMI before pregnancy exhibit a higher relative abundance of *Firmicutes* and a lower relative abundance of *Proteobacteria* compared with women of normal weight in their first trimester [4].

Pregnancy obesity can also affect a newborn’s microbiota. In fact, meconium samples of infants born to mothers who were obese pre-pregnancy showed fewer *Firmicutes* and increased Proteobacteria compared with those of infants born to mothers with normal pre-pregnancy BMI.

Additionally, in children’s gut microbiota, a higher maternal pre-pregnancy BMI was slightly correlated with a lower Shannon diversity index. Conversely, a child’s BMI z-score at five years of age was related to greater observed ASV (amplicon sequence variable) richness. Moreover, maternal pre-pregnancy BMI influenced the varying levels of several microbial ASVs in the *Ruminococcaceae* and *Lachnospiraceae* families [5,6]. 

## 3. Neurodevelopmental Disorders

Neurodevelopmental disorders represent a group of heterogeneous conditions with onset during the developmental age, characterized by an alteration of communication and social skills, learning, adaptive behavior, executive functions, and psychomotor skills. These deficits lead to an impairment of personal, social, scholastic, or work-related functioning. This new diagnostic category of the DSM-5 [7], in fact, compared with the previous category “Childhood and Adolescent Disorders” of the DSM-IV [8], underlines how these disorders are not limited to these age groups but tend to persist for a long time throughout life, changing according to the evolutionary trajectory.

These disorders often occur in comorbidity, representing a “constellation” of conditions that show overlapping clinical manifestations, probably because of common risk factors and shared pathogenetic mechanisms, defining a continuum between different pathologies (hypothesis of a genetic spectrum of neurodevelopmental disorders).

Neurodevelopmental disorders (DSM-5 [7]) include the following pathologies:

- Intellectual disability (intellectual development disorder);
- Communication disorders;
- Autism spectrum disorder;
- Attention deficit/hyperactivity disorder;
- Specific learning disorder;
- Movement disorders;
- Other neurodevelopmental disorders.

Neurodevelopmental disorders represent a very broad chapter, and the discussion of individual pathologies is beyond the purpose of this review.

## 4. Etiology and Risk Factors

Neurodevelopmental disorders are characterized by multifactorial etiology, consequent to a complex interaction between genetic and environmental factors (especially pre- and perinatal ones).

Examples of environmental factors in utero include exposure to alcohol, drugs, or other toxic substances during pregnancy, maternal stress, maternal infections, prematurity, and prenatal nutrition.

The genetic etiology has been widely confirmed: for example, as regards autism, the concordance of the pathology in homozygous twins is 60–90% and 5–40% in heterozygous twins [9]. The genetic factors include copy number variations (CNVs), single nucleotide variations (SNVs), and chromosomal abnormalities.

**Figure 1:**
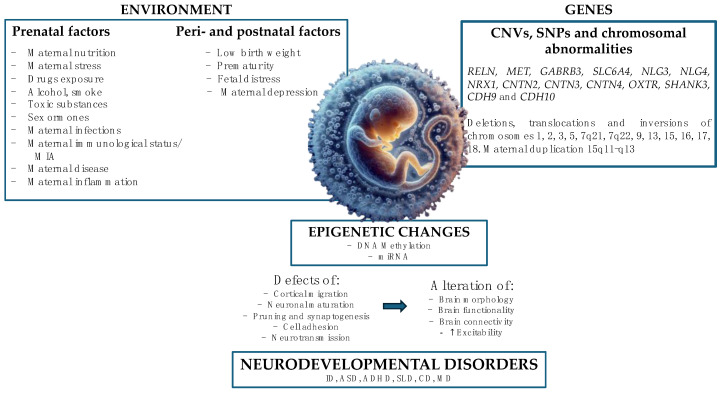
Interaction between environment and genes. Schematic illustration of the main environmental and genetic factors involved in neurodevelopmental disorders, based on literature review. Illustration by Federica Cernigliaro. (MIA: maternal immune activation; ID: intellectual disability; ASD: autism spectrum disorder; ADHD: attention deficit/hyperactivity disorder; SLD: specific learning disorder; CD: communication disorders; MD: movement disorders; perinatal factors act from 48 h before to 7 days after birth; postnatal factors act from 7 days after to 12 months after birth).

The interaction between these factors determines alterations in cortical migration and synaptic and neuronal networks, chromatin remodeling, regulation of transcription, and immunological regulation, constituting a trio of mutually interactive domains, therefore, environment, genes, and brain [10] [Figure 1].

The main risk factors for neurodevelopmental disorders, specifically for ASD, are as follows [11]:

- Complications of pregnancy and childbirth;
- Prematurity;
- Low birth weight;
- Exposure to alcohol, drugs, or other toxic substances during pregnancy;
- Low socio-economic level of parents;
- Situation of early emotional deficiency;
- High parental age (>35 years).

### 4.1. Genetic and Epigenetic Factors

The most recent studies show that CNVs and SNVs should be responsible for approximately 15% and 7% of ASD cases, respectively. Despite considerable progress, most ASD cases (>75%) still have unknown causes [12].

Genetic and epigenetic factors can act at various levels of the neuronal cell body through nuclear and cytoplasmic mechanisms [12], for example, chromatin remodeling, gene transcription, DNA methylation, post-transcriptional regulation by miRNAs, and many others.

The main genes involved in the etiopathogenesis of ASD code for molecular adhesion cells, scaffold proteins, proteins involved in neuronal communication, and voltage-gated ion channels [13], for example *RELN*, *MET*, *GABRB3*, *SLC6A4*, Neuroligins 3 and 4 (*NL*), Neurexins (*NRX*) 1 and 3, *SHANK3*, Cadherin (*CDH*) 9 and 10 (probably causing approximately 15% of ASD), and Contactin (*CNTN*) 2, 3, and 4. These mutations lead to impaired neuronal maturation and migration, reduction of apoptosis or increase in cell proliferation, alteration of cellular differentiation, reduction of dendritic spines and synaptogenesis (causing synaptopathies), resulting in alteration of brain morphology, functionality, and connectivity (reduced long-distance connectivity and excessive local connectivity) and excess excitability compared with neuronal inhibition. These alterations are the basis for neurodevelopmental disorders (Figure 1).

Genetics has a polygenic transcategorical role with vulnerability linked to neurodevelopmental disorders as a whole and not a single and specific disorder, suggesting the hypothesis of a neurodevelopmental continuum: there is, therefore, a complex overlap of genes involved in neurodevelopmental disorders [14]. 

Epigenetic mechanisms have the peculiar characteristic of modulating gene expression without altering the DNA sequence and represent the mediators of the gene/environment interface [15]. They can lead to modulation of susceptibility genes, causing alterations of brain morphology, functionality, and connectivity involved in neurodevelopment. In fact, epigenetic changes could be implicated in the stress susceptibility and pathogenesis of psychiatric disorders such as depression and schizophrenia, as well as neurodevelopmental disorders.

The main epigenetic mechanisms are as follows [12]:

- DNA methylation.
- miRNAs or microRNAs, small sequences of 20–25 RNA nucleotides, organized as a single strand, not coding for any protein but having the function of regulating the translation of target genes through downregulation mechanisms. They induce gene silencing through binding to complementary sequences on target mRNA molecules, resulting in repression of translation or degradation of the target molecule.

### 4.2. Environmental Factors

Environmental factors play a fundamental role in the pathogenesis of neurodevelopmental disorders. According to many recent studies, environmental factors should be responsible for approximately 40–50% of ASD [12] and include exposure to drugs or toxic agents, high parental age, nutrition, fetal environment, and many other factors.

High parental age is one of the most studied factors, although recently downsized, and would seem to be related to ASD, ADHD [16], bipolar disorder, and schizophrenia. A meta-analysis of 27 studies showed that a 10-year increase in maternal and paternal age was associated with a 20% greater risk of developing ASD, probably also due to age-related methylation changes in sperm. High parental age also appears to be associated with a reduction in the cortical thickness of the right posterior cingulate cortex in offspring with ASD.

Perinatal risk factors are widely studied and include birth trauma, umbilical cord complications, multiple births, maternal hemorrhage, low birth weight, neonatal anemia, genital malformations, incompatibility of the ABO or Rh system, and hyperbilirubinemia [17].

Other studies have also investigated the possibility of correlation with induced labor, cesarean section, prematurity (<36 weeks), and fetal distress.

Early life colonization could also have a pivotal role in neuropsychological disorders. In fact, immediately after birth, *Bifidobacteria* become primary microbial colonizers, establishing themselves as dominant genera in the gut of both infants and adults. These bacteria are potent probiotics with anti-inflammatory and antimicrobial properties [18]. *Bifidobacteria* also help reduce stress and alleviate depression by enhancing the hypothalamic-pituitary-adrenal axis response, increasing serotonergic precursor tryptophan, and exhibiting anxiolytic effects [19]. Deficiencies in intestinal *Bifidobacteria* have been linked to issues such as depression, particularly in individuals with neurodevelopmental disorders [20].

Additionally, studies indicate that early colonization with *Bifidobacteria* may impact neurodevelopment in early childhood. A low or absent abundance of *Bifidobacteria* could be associated with vulnerability and immaturity [21]. *Bifidobacterium* spp. play a crucial role in healthy infant gut microbiota development and immune system regulation and may exert neuroprotective effects by modulating neuroactive metabolite production and release [22].

Different studies have also shown that the early shaping of the microbiome plays a crucial role in microglial maturation and modulates glial activation in the central nervous system. This modulation is considered a key factor in regulating neuroinflammation within the CNS [23,24], eventually predisposing to the onset of neurodevelopmental disorders such as ASD, ADHD, and schizophrenia [23]. Future research could focus on therapeutic interventions and preventative strategies that leverage early microbiome health to mitigate or even prevent such conditions, potentially transforming approaches to neuropsychological disorders from infancy.

Another potential risk factor for ASD is fetal exposure to sex hormones (in fact, the fetal testosterone theory was one of the theories proposed to explain the major prevalence of ASD in males, a very controversial hypothesis) [25]. Some studies have detected high levels of sex hormones and cortisol in amniotic fluid samples from male autistic patients compared with controls [26]. The same authors reported an association between fetal levels 31 of estrogens, important for synaptogenesis, and the risk of autism. Other studies have found post-mortem reduced levels of estrogen beta receptors and aromatase in the frontal gyrus in ASD patients compared with controls. Furthermore, several SNPs of protein-coding genes involved in the synthesis or transport of sex hormones appear to be associated with autistic traits.

Maternal health conditions have a high impact on the risk of ASD. Maternal nutrition is essential for correct fetal development; nutrition can cause DNA methylation, leading to epigenetic regulation of important genes involved in neurodevelopment. Prenatal and postnatal exposure to different nutritional elements or deficiencies can induce persistent brain alterations, predisposing to neurodevelopmental disorders. So, maternal nutrition is crucial in the prevention of brain disorders, as we will see subsequently. 

Maternal drug use, smoking, and alcohol during pregnancy constitute potential risk factors for ASD. Smoking and alcohol would appear to have a weak association with ASD in cases of mild or moderate consumption [27,28]. Regarding drug use, it is difficult to establish the safety of drugs during pregnancy. Valproic acid [29,30] (antiepileptic and mood stabilizer) and selective inhibitors of serotonin re-uptake—SSRIs—(antidepressants) definitely should not be used: the first one is associated with congenital malformations, developmental delay, and cognitive alterations; the second one appears to be associated with a 50% increased risk of ASD.

Maternal diseases can also represent a risk factor: case-control studies showed a 62% increased risk of developing ASD in diabetic mothers compared with non-diabetics, while another study found a 74% increased risk for pregestational diabetes and 43% for gestational diabetes [31].

Maternal infections are associated with an increased risk of ASD, especially due to the immune response; in fact, agents of the complex TORCH are related to postnatal brain dysfunction, leading to neurological disorders [32].

Maternal immunological status is pivotal during fetal development. Studies reported an association between family history of autoimmune disease and ASD [33]. Zinc could play a role in regulating the immune system and in the formation of the neural tube. Maternal immune activation (MIA) during pregnancy has been proposed as a potential etiological cause of impairment of synaptic pruning and microglial-mediated neurogenesis. Different clinical studies suggest that the pro-inflammatory profile during maternal obesity is associated with an increased risk of developing ASD [34].

Such pro-inflammatory assets, derived from an epigenetic program of immunity, promote micro- and macrostructural anomalies of the brain in the offspring, maybe lasting until adulthood, accompanied by phenotypic changes [35]. Moreover, there is a cross-talk between the central nervous system and the immune system, probably leading to macro- and microstructural brain defects, increasing the susceptibility to ASD.

During CNS development, microglia plays the role of antigen-presenting cell [23], but also regulates neurogenesis and synaptic plasticity [36]. Therefore, an hyperactivation of the immune system at this stage could cause deleterious effects on brain development, leading to disorders such as epilepsy, schizophrenia, Parkinson’s disease, Alzheimer’s disease, and ASD [37].

Therefore, maternal inflammation represents an important stress factor for fetal development, and it can be determined by various conditions, such as infections, but also maternal nutrition [38]; in fact, pro-inflammatory foods and diets determine a chronic inflammatory state, regardless of BMI.

Finally, another potential risk factor is fetal exposure to toxic xenobiotics, some of which can cause mitochondrial toxicity (documented in some patients with ASD) with alteration of cerebral energy balance [39].

Toxic agents include the following:

- Heavy metals can alter many body functions, causing neurological and behavioral damage, which we will analyze later. A meta-analysis found a 60% increased risk of ASD due to exposure to high levels of inorganic mercury [40].
- Organophosphates are associated with a 60% increased risk of developing ASD [41]. This category includes non-persistent organic pollutants (phthalates and bisphenol A) and persistent organic pollutants (DDT, PCBs, and PBDEs). Exposure to these substances seems to alter calcium-related signaling pathways, leading to alterations in dendritic growth and anomalies in neuronal connectivity, both typical of ASD.

Moreover, all these risk factors can interact with each other, influencing fetal neurological development.

Environmental factors, especially heavy metals, can affect neurodevelopment through various mechanisms [42]:

- Epigenetic/genetic mechanisms (discussed previously).
- Immune dysregulation and neuroinflammation—in fact, the interaction between the immune system and nerve cells is essential for neurodevelopment. For example, IL-6 and INF-γ regulate dendritic growth and synaptogenesis through signal transduction mechanisms and the MAPK pathway.
- Oxidative stress and mitochondrial dysfunction through an imbalance between free radicals and antioxidants leading to the alteration of ATP levels in nervous cells.
- Endocrine alterations, such as hormonal imbalances.
- Alterations of neurotransmitters (such as serotonin, glutamate, and GABA) and cell signaling pathways.

## 5. Prenatal Nutritional Factors

The importance of maternal nutritional factors gained attention during the 20th century, first with the study about the role of folic acid supplementation in reducing and preventing neural tube defects [43], and subsequently with the discovery that low levels of iron were associated with preterm birth, maternal mortality, and developmental alterations [44].

Therefore, we know that preconceptional and prenatal nutrition is essential for fetal brain development.

A 2019 review [45] investigated 2169 articles, evaluating the association between preconception and prenatal nutrition and neurodevelopmental disorders, such as ASD, ADHD, and ID: 20 articles on ASD and 17 on ADHD were analyzed, showing an inverse association between maternal multivitamin and folic acid supplementation and the risk of ASD in offspring.

Maternal nutrition can modulate gene expression through epigenetic mechanisms; for example, the methyl-donor micronutrients of the one-carbon metabolism, such as folic acid, vitamin B6 and B12, and choline, play important roles in many physiological pathways and processes, including DNA methylation. So these micronutrients seem to alter gene function during critical stages of brain development, leading to susceptibility to neurodevelopmental diseases [46].

Exposure to an unbalanced diet in early life and during the life span negatively modulates gene expression, leading to epigenetic changes associated with neurodevelopmental disorders and neurodegenerative diseases later in life [47].

Maternal nutrition plays a fundamental role in various phases:

- The maternal diet before pregnancy allows to optimize nutritional status in order to maintain a healthy pregnancy and support fetal development [48].
- Maternal nutrition during the conception period is important for gametes’ function and health and for placental development [49]. For example, the first 2–3 weeks from conception represent a period of rapid development in which the embryo undergoes multiple processes (neuronal migration, synaptic formation, apoptosis) for fetal brain development, constituting a highly sensitive period to possible environmental disturbances that could predispose the fetus to develop neurodevelopmental disorders in the postnatal period.

Inadequate nutrient intake during pregnancy is associated with brain defects, a greater risk of behavioral and neuropsychiatric disorders, cognitive and visual alterations, and motor deficits.

The main neurodevelopmental outcomes described in the literature include the following [50]:

- Behavioral and psychiatric disorders (ASD, ADHD, anxiety, depression, schizophrenia);
- Cognitive alterations (intellectual disability, language disorders, learning, and memory disorders);
- Neural tube defects;
- Visual alterations;
- Motor deficits;
- Neural molecular dysfunctions (synaptic alterations, neurotransmitters’ metabolism, myelination, cell differentiation, axonal growth, dendritic arborization, anti-inflammatory regulation, oxidative stress, death neuronal, vascular function, synaptic plasticity, neurogenesis, astrocytic alterations);
- Structural changes (reduction of brain volume, spina bifida, hydrocephalus, alteration of the hypothalamic and hippocampal pathways).

A scoping review of 2021 [50] analyzed 84 studies in order to investigate the association between maternal nutrition during pregnancy and neurodevelopment in offspring, summarizing data about the role of main maternal nutritional factors. This review focused on these nutritional categories:

- Vitamins and minerals, such as vitamins B12, A, E, D, K, folic acid, iron, copper, creatine, choline, zinc, and iodine;
- Macronutrients such as polyunsaturated fatty acids (PUFAs) and proteins;
- General maternal nutritional status, such as obesity, overnutrition (high fat–HFD—and hypercaloric diets), and undernutrition;
- Other nutrients, such as gangliosides and caffeine.

Also, heavy metals have to be mentioned because of their possible presence in food, with a negative impact on health, especially regarding the offspring.

Maternal supplementation of nutrients in the preconception and prenatal period provides not only the foundations for optimal fetal brain development but may also be able to “program” the brain through epigenetic mechanisms and determine the risk or degree of resistance to certain neurological conditions throughout life.

In fact, maternal nutrition intervenes in signaling mechanisms, causing epigenetic remodeling of fetal genes and influencing placental development and nutrient transfer [51].

### 5.1. Dietary Habits

Maternal malnutrition includes conditions of overnutrition such as high-fat diets (HFD) and hypercaloric diets, but also situations of undernutrition characterized by nutritive restriction. All these conditions determine alterations in neuronal excitability and brain development due to a reduction of hippocampal neurons, leading to a susceptibility to neurodevelopmental disorders [52].

#### 5.1.1. Undernutrition

The fetal brain consumes 75% of fetal energy (mainly glucose), so its development is susceptible to nutrient restriction.

An insufficient nutrient intake causes different consequences depending on the phase of pregnancy: at the beginning, it causes alterations in neuronal proliferation; at the later stage, it causes alterations in neuronal differentiation [53].

Protein restriction during pregnancy, however, causes alterations in neuronal differentiation, mitochondrial function, fetal astrocytogenesis, and apoptosis [52].

Maternal undernutrition seems to determine epigenetic changes in the fetal brain related to an increased expression of genes coding for the glucocorticoid and proopiomelanocortin receptor at the level of the fetal hypothalamus, leading to increased activity of the hypothalamic-pituary-adrenal axis (HPA) [52].

#### 5.1.2. Overnutrition

Maternal high-fat diet (HFD) causes neural effects on both structural and functional levels, resulting in reduced brain development. These effects include increased proliferation in the hypothalamus but also a reduction in apoptosis and neuronal differentiation in the dentate gyrus in animal models [53].

Maternal HFD seems to induce alterations of serotonergic and dopaminergic systems in the nucleus accumbens and ventral tegmental area, leading to an increased risk of neurodevelopmental and behavioral disorders [54]. Moreover, maternal HFD increases levels of inflammatory cytokines in offspring, altering neural development.

A maternal hypercaloric diet seems to cause the reprogramming of myeloid progenitor cells, increasing the immune response throughout life [54].

HFD leads to the alteration of the brain reward circuit (nucleus accumbens, ventral tegmental area, prefrontal cortex) through the hypomethylation of opioid gene promoters, increasing the obesity risk in offspring [54].

Maternal immune activation induced by HFD (diet-induced MIA) determines a chronic pro-inflammatory profile during fetal development, leading to hyperactivation of microglia [35]. The cross-talk between the immune system and CNS induces epigenetic modifications on immune cells in offspring, such as DNA hypermethylation and aberrant expression of proinflammatory genes in the CNS, responsible for micro- and macrostructural defects in several brain regions with alterations of fetal synaptic pruning and neurogenesis, leading to susceptibility to autism.

Maternal obesity during pregnancy could induce immune-inflammatory modifications, altering fetal neuronal pathways involved in the regulation of behavior and cognitive performance. Among the various mechanisms, there is an increased expression of glucocorticoid receptors at the hippocampal level with changes in HPA axis activity and an increased expression of fetal cytokines, such as IL-6, related to working memory dysfunctions and dementia [55]. Epigenetic changes intervene on the dopamine and serotonin pathways, critical in the regulation of behavior [55]. There are inflammatory and neuroendocrine changes.

Some studies showed that maternal overweight appears to increase the risk of ADHD by 30%, ASD by 10%, and intellectual developmental delay by 23%. Obesity before pregnancy seems to determine an increased risk of developing neurodevelopmental outcomes by 17% [55]. According to various clinical studies, maternal obesity is responsible for 1.39–1.59% of ASD cases.

A meta-analysis showed a 28% increased risk of ASD in the offspring of overweight mothers and 36% in the offspring of obese mothers, with an increased risk of ASD even in the case of maternal underweight [56].

Some studies suggested that exposure to sucrose induces hyperactivity, impulsiveness, and attention disorders, secondary to alterations in the transport and expression of striatal dopamine. High maternal consumption of fructose is related to metabolic syndrome and obesity with increased hyperexcitability and alteration of prefrontal cortex function in offspring (as in ASD patients) [57]. In animal models, there was also a hippocampal cognitive impairment due to hypermethylation and reduced expression of the BDNF gene.

High leptin levels in obese mothers are associated with placental dysfunction and alteration of neural development in offspring [54]. High levels of leptin, insulin, and ghrelin have been found in children with ASD; these hormones cross the blood–brain barrier and regulate neuronal plasticity through the facilitation of glutamatergic and GABAergic activity, which promotes cognitive processes.

Maternal obesity is related to gestational diabetes, hyperglycemia, and hyperinsulinemia, which can cause alterations in neuronal circuits during the period of brain development [54]. In mice, insulin resistance seems to lead to reduced neurogenesis and synaptic plasticity. Maternal obesity could be related to structural alterations, such as anencephaly and spina bifida; however, current studies are insufficient to establish an association between maternal BMI and brain structural anomalies.

### 5.2. Nutrients

During pregnancy, macronutrients cross the placenta, through various placental transport systems, in order to support fetal growth [58], so an adequate intake of various macronutrients is crucial for optimal fetal development.

Furthermore, various macronutrient requirements undergo variations during pregnancy: in the first phase of gestation, glucose provides 80% of the energy, the fatty acids are maternally derived, and the proteins are used for fetal growth; during the late gestation, glucose provides 80% of the energy, but there is an increased demand for fatty acids; in the early postnatal phase, blood glucose drops and fatty acids provide 50% of the energy [58].

Table 1 summarizes the alterations and functions of the main nutrients during prenatal phase, based on literature [45,50].

**Figure 2:**
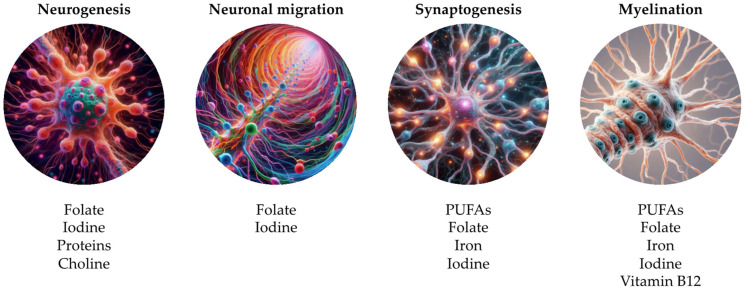
Alterations of the main nutrients during prenatal phase, based on literature [45,50]. Illustration by Federica Cernigliaro. (PUFAs: polyunsaturated fatty acids).

Figure 2 shows the alterations of the main nutrients during prenatal phase, based on literature [45,50].

#### 5.2.1. Fatty Acids

Polyunsaturated fatty acids (PUFAs), especially arachidonic acid (AA) from the omega-6 family and docosahexaenoic acid (DHA) from the omega-3 family, are structural components of the phospholipids of the neuronal membrane and myelin sheath. So, PUFAs play an important role in the development of the fetal brain, NS, and retina from the 24th week of gestation until childhood [58].

The maternal integration of DHA and PUFAs is essential for placental function, neuronal synaptogenesis, and dendritic growth, and the fetus is dependent on the mother’s PUFAs until 16 weeks after birth [59].

A low intake of omega-3 decreases DHA levels in the brain, leading to an alteration of neurogenesis and neurotransmitter metabolism, especially dopamine and serotonin, and behavioral dysfunction in animal models.

Some studies have analyzed the effects of DHA deficiency on the modulation of GABA-ergic receptors, showing an increased activity of PLA2 (phospholipase A2) in the plasma membrane, leading to inhibition of the GABA-A receptor and increased neuronal excitability [60].

Furthermore, a reduced intake of omega-3 in mice seems to lead to decreased levels of dopamine in the frontal cortex, increased release of serotonin in basal synaptic, and modification of the glutamatergic system in offspring [61]. Moreover, DHA supplementation appeared to increase synaptic plasticity in hippocampal neurons and improve glutamatergic neurotransmission. However, despite DHA supply, its levels increase differently in different regions of the brain: the hippocampus and frontal cortex need a longer time to restore normal DHA levels after prenatal deficiency; therefore, it may be difficult to restore DHA levels in the absence of postnatal dietary intervention.

A deficiency of omega-3 also leads to a decreased development of the hippocampus due to the alteration of post-mitotic cell migration and a reduced size of neuronal cell bodies at the level of the parietal cortex and hypothalamus [50].

A Western diet rich in meat and processed foods, containing omega-6 (pro-inflammatory), and low consumption of seafood, containing omega-3 (anti-inflammatory), such as DHA, constitute a risk factor for neurodevelopmental disorders. In fact, high levels of DHA appear to reduce the risk of schizophrenia, bipolar disorder, depression, and anxiety. So, a correct balance between omega-6 and omega-3 is essential [59].

Moreover, fatty acids can induce the methylation of BDNF, nerve growth factor, and vascular endothelial growth factor, influencing fetal development.

Today the recommended intake of DHA in pregnant women is 200 mg/day. Children should also have an adequate intake of PUFA during their development.

#### 5.2.2. Proteins

Proteins are crucial during the second and third trimesters because they support the increased growth of fetal tissues. Moreover, proteins are an alternative source of energy in cases of carbohydrate deficiency [50]. Protein intake seems to have positive effects in terms of motor and cognitive skills [62].

During pregnancy, protein requirement increased by 1 g/day in the first trimester, 8 g/day in the second trimester, and 26 g/day in the third trimester.

Data recommends a daily intake of 45 g and 50 g of protein during pre-pregnancy and pregnancy, respectively.

Protein deficiency leads to alterations of hippocampal neurogenesis, decreasing BDNF and IGF, and reduction of brain and neuronal volume. Another effect is the reduction of maternal lipid availability and fetal brain fatty acid levels, leading to the possible reduction in myelin production and increasing the risk of neurodevelopmental disorders [58].

#### 5.2.3. Folates

Folates play a role in many metabolic reactions and pathways, being the precursor of amino acids and nucleic acids.

A decreased folate intake leads to an alteration of brain development due to incorrect DNA methylation [45].

The role of preconception and prenatal supplementation of folic acid, the synthetic form of folates, in the prevention of neural tube defects has been widely described and studied in the literature, as has its importance in the processes of neuronal proliferation, migration, differentiation, vesicular transport, and synaptic plasticity; in fact, in mouse models, folic acid promotes neurogenesis and has axonal regeneration.

Several studies highlight the role of folic acid in cognitive functions and learning [15]. Furthermore, an association between maternal folate intake and increased risk of ASD has been described, with the identification of the MTHFR 6777 C > variant T [15].

Data recommend a periconceptional supplementation of 400 mg/day [63].

#### 5.2.4. Iron

Iron requirements increase during pregnancy, so prophylactic iron supplementation of 30–40 mg/day from the 20th week of gestation could support and regulate the neuronal energy metabolism during fetal development [63].

Crucial processes such as myelination, synaptogenesis, dendritogenesis, and neurotransmission depend on iron-containing enzymes and hemoproteins.

A martial deficit during the first trimester can lead to alterations in gray matter and dendritic structure, but also in the fetal hippocampus, resulting in impaired memory and learning, as well as in the risk of social dysfunction, low cognitive performance, reduced linguistic skills, and behavioral alterations [63]. These outcomes persist despite the postnatal martial supplementation.

Iron excess can also be harmful to the fetus due to its ability to generate reactive oxygen species, resulting in tissue damage [63].

#### 5.2.5. Iodine

Iodine is essential to synthesize thyroid hormones, which are fundamental in the first trimester of gestation, especially maternal T4, which is essential for neuronal migration and myelination of the fetal brain. Its absence leads to irreversible neurological damage [64]. Therefore, iodine deficiency determines altered fetal neurogenesis, neuronal migration, synaptogenesis, and myelination. Data describe neurological outcomes such as congenital anomalies, cognitive and motor deficits [64], and endemic cretinism; in fact, iodine deficiency is the main cause of intellectual disability worldwide.

Scientific evidence supports iodine supplementation during pregnancy (suggested intake of 250 mg/day and consumption of iodized salt).

#### 5.2.6. Copper

Copper is an essential element, important in several biological processes, and necessary for normal fetal development. Copper supplementation during pregnancy has not been examined yet, but in animal models, copper deficiency leads to death of the embryo or structural, biochemical, neurological, and immunological anomalies [65].

In excess, copper is also a suspected neurotoxicant due to its highly reactive nature and its ability to cause oxidative stress [66].

Moreover, copper deficiency in pregnancy seems to lead to an alteration of perinatal development [66].

One study identified a particular association between ADHD and copper, showing a U-shaped pattern with higher risk at both lower and higher levels. Conversely, a study about ADHD symptoms in children found no association with copper levels during pregnancy [67].

No studies have been conducted as regards the possible association between prenatal copper levels and neurological outcomes (cognitive, linguistic, and motor functions). However, recent cross-sectional studies reported an association between higher copper levels during childhood and increased risk of ADHD [68]. Furthermore, studies both on human and animal models suggest that prenatal copper toxicity may contribute to ASD.

#### 5.2.7. Creatine

The daily requirement of creatine is approximately 2 g in adults. Creatine can be obtained through animal-derived products or supplementation, while in the child through breastfeeding or formula milk [50]. Creatine is also synthesized 47 endogenously from the liver. It has an important role in maintaining cellular levels of ATP and in stabilizing the mitochondrial membrane potential. In some studies on mouse models, creatine supplementation in mice with fetal asphyxia seemed to mitigate motor alterations.

However, there are still few studies regarding neurological outcomes and creatine supplementation in pregnant women.

#### 5.2.8. Choline

Choline, at an exogenous level, is found in animal-derived foods and, to a lesser extent, of plant origin. It can also be produced endogenously in the maternal liver. Choline deficiency is common; an estimated 90% of pregnant women could suffer from choline deficiency in the USA.

This micronutrient plays a role in various pathways, including the synthesis of phospholipids and neurotransmitters. It acts as a methyl group donor, resulting in epigenetic modifications in the fetal brain and placenta; moreover, it is involved in the proliferation of stem cells and transmembrane signaling during neurogenesis [62].

Choline also has an important role in neurodevelopment, being implicated in adaptive modulation of cognitive functions. Therefore, maternal choline deficiency alters neurogenesis and angiogenesis in the fetal hippocampus [52]. According to some studies, oral integration of choline in the second trimester and after birth seems to be associated with better sensorial gating [52].

Some studies suggest that maternal intake and maternal choline concentration are inversely proportional to the risk of neural tube defects in offspring.

#### 5.2.9. Zinc

It is estimated that approximately 30% of the global population has a mild-moderate zinc deficiency [69]. Zinc plays an important role in fetal development, intervening in the metabolism of carbohydrates and proteins, synthesis of nucleic acids, and cellular division and differentiation [69]. Moreover, it seems to have an important regulatory role in the immune system.

Studies on mouse models indicate that gestational zinc deficiency is associated with a decreased mass in the cerebellum, limbic system, and cerebral cortex, with a reduction in cell number [70].

Many studies showed an association between low levels of zinc and the risk of ASD or developing ASD-related symptoms, but also the “therapeutic” role of zinc integration [71].

Low levels of zinc or an alteration of the zinc/copper ratio have been found in the hair and serum of children with ASD [71]. Prenatal zinc deficiency in mouse models determines alterations in social behavior.

In vivo and in vitro studies have demonstrated that a zinc deficiency at the synaptic level determines a reduction of some members of the ProSAP/Shank family and synaptic density, leading to ASD-related symptoms in mice.

Furthermore, maternal zinc supplementation appears to prevent neural tube defects in offspring.

#### 5.2.10. Vitamin B12

Vitamin B12 is mainly contained in products of animal origin, so vegan and vegetarian diets may increase the risk of B12 deficiency during pregnancy. Sufficient levels of this vitamin guarantee normal neuronal development and adequate myelination [70].

It acts as an enzyme and as a cofactor and is essential for the metabolism of fats and proteins, for hemoglobin synthesis, and contributes to DNA methylation.

Some case reports show an association between deficiency of this vitamin during pregnancy (values below 200 pg/mL) and irritability, reduced brain growth, and increased risk of neural tube defects [63].

#### 5.2.11. Vitamin B6

Vitamin B6 is contained in foods of animal origin, but also vegetables. It plays an important role as a coenzyme in the metabolism of amino acids, carbohydrates, and fats, and it is important for the correct development and maintenance of the CNS and immune system. Moreover, it is essential for serotonin synthesis and involved in regulation of mood, sleep, appetite, memory, and concentration skills. In addition, vitamin B6 plays a key role in regulating methylation mechanisms, influencing gene expression, and various pathways involved in neurodevelopment and neurological functions [46].

#### 5.2.12. Vitamin D

Vitamin D can regulate gene expression [52], influencing brain development. Currently, the recommended intake during pregnancy is 600 UL/day.

Prenatal vitamin D deficiency causes brain development abnormalities and alterations of neuronal differentiation, neurotransmission, various pathways, and calcium homeostasis, as well as post-translational modifications, underscoring the role of this vitamin in epigenetic regulation [52].

Recent studies show a correlation between prenatal vitamin D values and increased risk of ADHD and ASD, or alterations in cognitive and linguistic development [72].

#### 5.2.13. Vitamin A

Retinoic acid, or vitamin A, plays a role in neuronal differentiation, axonal growth, and organogenesis, as well as in the regulation of the expression of developmental target genes.

In pregnant women, an intake of 600 mg/day is recommended [50], remembering that high levels of vitamin A (>1000 IU/day) have teratogenic effects.

#### 5.2.14. Vitamin E and K

There is currently little evidence regarding the role of maternal integration of these vitamins for fetal neurological development.

#### 5.2.15. Gangliosides

Gangliosides constitute 6% of the phospholipids in the SN and play a crucial role in brain development, being involved in neuronal repair, signal transduction, and myelinization. There are various subtypes of gangliosides (GM1, GD1a, GD1b, GT1a, and GT1b) present in different concentrations during neuronal proliferation and maturation [73].

According to some studies, pregnancy supplementation with gangliosides could enhance the cognitive development of offspring [73].

During fetal development, they are found mainly in the hippocampal region, with cognitive and memory functions.

#### 5.2.16. Caffeine

During pregnancy, caffeine metabolism is reduced, especially after the first trimester, and its half-life increases from 2.5–4.5 h up to 15 h [74]. Furthermore, caffeine is a lipophilic substance, so it is able to cross the placenta, but the fetus does not have the enzymes for its metabolism. For this reason, caffeine can alter gestational health and fetal neurodevelopmental. Additionally, caffeine absorbed by the mother can accumulate at the level of the uterine environment, where it can potentially act at the level of embryonic development and generate adult-onset diseases.

The mechanisms by which caffeine determines adverse outcomes are not well known, but we know that this substance and its metabolites (paraxanthin, theophylline, and theobromine) are non-selective antagonists of adenosine, acting through the blockade of adenosine receptors, which are the first receptors expressed at the level of the embryonic brain, and their modulation can potentially alter axonal growth.

Epidemiological studies show a correlation between caffeine consumption during pregnancy and intrauterine growth restriction (IUGR) or low birth weight, miscarriage, microcephaly, childhood overweight, and cognitive disorders [74].

The dose of caffeine considered safe for gestational health is a daily intake less than 300 mg (about three cups of coffee) [75].

Some animal studies have demonstrated an association between maternal elevated consumption of caffeine and alteration of sleep, locomotion, and learning, as well as anxiety and behavioral alterations [76].

Human studies have reported an increased risk of ADHD in children exposed to more than 10 cups of coffee per day during pregnancy, while a lower maternal intake was not found to be related to any risk [45]. However, another study found a threefold increased risk of ADHD following intrauterine exposure to 10 or more coffee, but the risk became statistically insignificant due to the correction of confounding factors, with the conclusion of a lack of association between high caffeine consumption and ADHD [76].

Another study demonstrated that an intake of more than 200 mg/day of caffeine during pregnancy is associated with a twofold increased risk of cognitive alterations and IQ impairment at 5.5 years, compared with children exposed to doses lower than 100 mg/day [77].

Further studies are needed to confirm the association between high consumption of caffeine during pregnancy (>1000 mg/day) and neurodevelopmental disorders.

### 5.3. Heavy Metals

Prenatal exposure to heavy metals represents a risk factor for neurodevelopmental disorders [42] because fetal NS development constitutes a critical phase of rapid growth, characterized by greater vulnerability towards toxic agents compared with adults [45].

Moreover, the fetus is extremely vulnerable to environmental toxic substances also due to their ability to cross the placental barrier; for example, heavy metals such as lead and mercury can reach the fetus during pregnancy. The blood–brain barrier does not form until 6 months after birth, so prenatal exposure to toxic substances causes major damage to the brain, from subclinical dysfunction to various neurodevelopmental disorders, depending on the level of exposure. This exposure, combined with genetic predisposition or epigenetics, can alter normal neurodevelopmental processes.

Therefore, there could be a correlation between the level of environmental toxic agents and the increasing number of diagnoses of neurodevelopmental disorders such as ASD [42].

Prenatal exposure to heavy metals could derive from maternal environment exposure or maternal nutrition during pregnancy.

One study showed that exposure to diesel, mercury, lead, chloride, methylene, and manganese during the perinatal period is closely linked to ASD [78]. A research group in Saudi Arabia found increased levels of mercury and lead in the blood and a notable decline in selenium in ASD children compared with neurotypical children [79].

Furthermore, low levels of glutathione (GSH) have been detected in patients with ASD, suggesting a possible link with the disorder [80]. GSH is an antioxidant, which constitutes the main defense mechanism against free radicals, and heavy metals may alter its production.

A 2021 study [67] measured maternal blood concentrations of 11 metals/elements (cadmium, cesium, cobalt, copper, lead, magnesium, manganese, selenium, zinc, arsenic, and mercury) at the 17th week of gestation to evaluate a possible association with ASD and ADHD. The results showed several associations, especially regarding arsenic, cadmium, copper, mercury, manganese, magnesium, and lead, suggesting negative effects of these metals on neurological development and a correlation with the two pathologies. For ADHD, negative associations were found with mercury, while positive associations (increased risk) were found with cadmium and magnesium. For ASD, negative associations were found with cesium, copper, mercury, and zinc, while positive associations (increased risk) were found with cadmium and manganese. Therefore, some negative and positive effects can probably cancel each other out. However, in some populations exposed to greater quantities of toxic metals and/or variations of essential elements, it is possible a greater impact on neurodevelopmental outcomes.

However, there is a lack of studies investigating toxic metals, essential elements, and neurodevelopment using mixed approaches. Toxic metals can, in combination with other metals and various nutrients, including essential elements, produce interactive effects (additive, synergistic, or antagonistic), with a negative impact on neurodevelopment, without the possibility of predicting the net effect by analyzing only individual compounds. So, further studies and investigations could allow us to analyze the combined effects of metals/elements and their mechanisms of action.

Table 2 resumes the main pathogenetic mechanisms and alterations induced by heavy metals during prenatal phase, based on literature [42,67].

#### 5.3.1. Lead

Lead is a non-essential metal that is abundant in the surrounding environment, with various industrial and domestic uses.

Excessive lead exposure in children is associated with nervous system damage with negative effects on neurodevelopment, probably already at levels below 10 μg/dL [81]. Through mechanisms not yet known, lead seems to compromise normal neurological development, leading to cognitive deficits in adult life and violent/criminal behavior.

Various studies have analyzed lead exposure as one of the causes of ASD [82]. One study showed a high level of Pb and Hg and a low level of antioxidants (GSH and vitamin E) in the blood of ASD patients compared with neurotypical children [83]. Different other studies highlight elevated blood levels of Pb in ASD compared with neurotypical controls. Elevated Pb levels were linked to abnormal behavior, communication deficits, and emotional dysregulation [84]. A recent study showed that Pb induces dopaminergic dysfunction in the NS, with structural damage to *Caenorhabditis elegans* (*C. elegans*) neurons [81]. Abnormal dopamine signaling led to functional consequences with behavioral alterations. The study also showed an alteration of the dopamine transporter and a reduction of dopamine levels associated with monoamine oxidase inhibition.

Interestingly, the dopaminergic dysfunction has been associated with autistic-type behaviors, with the involvement of both mesolimbic and nigrostriatal circuits.

#### 5.3.2. Mercury

Mercury (Hg) occurs naturally in the environment and has become increasingly abundant due to human activities, with a 1.5% increase in atmospheric Hg. Mercury is mostly transferred into the organic system through contaminated foods, such as plants or fish, and exists both in organic and inorganic form [85].

Overexposure to mercury leads to crossing the blood–brain barrier (BBB) with accumulation in the cerebellum, visual cortex, and spinal cord [86].

There are conflicting studies on the possible association between mercury and ASD. Some studies have compared the amount of Hg in the blood of pregnant women and have not found autism-related behaviors in offspring [87]. Instead, another study has found a notable Hg increase in the brain and blood of ASD patients compared with neurotypical controls [86].

Some mechanisms are responsible for the elimination of substances; for example, glutathione-S-transferase (GST) can eliminate foreign substances and drugs. Another mechanism involves the attachment of mercury to GSH for excretion through the bile.

Various studies have shown that these mechanisms are dysfunctional in ASD patients with ASD, leading to increased levels of Hg in the tissues. Hg accumulation causes oxidative stress, brain inflammatory reactions, and autoantibodies increase, needed for the onset of autism and other neurodevelopmental disorders [86]. Another study reported a positive relationship between maternal exposure to mercury fillings and ASD in offspring [88]. Additionally, various studies have evaluated blood and hair samples of ASD children and have linked fetal and postnatal exposure to Hg and/or Pb to autistic traits [67]. Interestingly, the study also found that the use of anti-heavy metal treatment alleviated the autistic symptoms. In contrast, many other studies have shown no association between Hg and ASD.

Moreover, mercury poisoning and autism have elements in common, including neuronal necrosis, dendritic overgrowth, gliosis, mitochondrial dysfunction, neuroinflammation, brain immune response, imbalance between free radicals and antioxidants, lipid peroxidation, and axonal demyelination [89].

However, some of these studies present a confounding factor, or fish and seafood maternal intake; further studies are therefore necessary to distinguish the potential negative impact of mercury exposure resulting from fish consumption on neurological development by the positive effect of beneficial nutrients coming from the same source.

#### 5.3.3. Cadmium

Cadmium can have direct or indirect effects on brain development: it interferes with the progression of the cell cycle, proliferation, differentiation, and apoptosis.

There is little information on the possible relationship between cadmium exposure and the increased risk of autism. Studies have recorded a significant decrease in Cd level in the hair of children with autism compared with neurotypical children [90]. However, other studies contradict these findings. Another study [91] has reported a decrease in the concentration of Cd in the urine in children: the reduced urinary elimination of Cd could be compatible with the alteration of the mechanisms of degradation of metals, with consequent increase in blood levels.

In 1967 [92], the evaluation of hair metal levels of autistic children (aged 0–15 years) found that 8.5% of patients had an increase in Cd concentration. Specifically, the highest concentration of Cd (12.1%) was found in children aged between 0 and 3 years in conjunction with the reporting of Pb. These findings suggest a vulnerability to poisoning by environmental toxic substances during the first years of life, which can lead to epigenetic changes and neurodevelopmental disorders.

Some studies [92] have reported the preferential accumulation of these metals in females, with a likely higher rate of susceptibility during pregnancy.

#### 5.3.4. Manganese

Manganese is an essential metal and has various physiological functions, including homeostatic functions, degradation of proteins, lipids, and carbohydrates, brain activity, and enzyme cofactor [93]. However, it has been reported that excessive exposure to Mn induces neurotoxicity and could also be an environmental risk factor for neurodegenerative diseases [94]. Studies found associations between excessive exposure to Mn and cognitive impairment, short-term memory loss, and attention deficit.

Childhood/maternal exposure to Mn can occur through house dust, contamination via vehicle emissions, industrial waste, and tap water, causing neurotoxicity [95].

The levels of some metals, including Mn, in tap water have been studied in the house dust and environmental soil, finding a high level of Mn. So, early Mn has negative effects on motor skills and cognitive abilities.

Many research studies have investigated the role of Mn in the etiology of ASD using the quantitative assay of Mn level in hair, blood, urine, enamel tooth, and in the atmosphere, reporting variable results. One study [96] documented an inverse relationship between Mn level in the blood and the concomitant degrees of mental development. Another study [97] supported an inverse association between Mn and autistic traits. They also compared Mn levels in the tooth enamel, finding a significantly lower concentration in ASD patients compared with neurotypical children [98]. In contrast, no difference was also documented in Mn concentration in the hair of ASD patients compared with the control group.

Overexposure to Mn causes an imbalance between reactive oxygen species and antioxidants [99]. GST genes are responsible for coding the enzymes involved in the regulation of the level of free radicals. These enzymes include glutathione S-transferase mu 1 (GSTM1), glutathione S-transferase theta 1 (GSTT1), and glutathione S-transferase pi (GSTP1).

One study [99] suggests that an alteration of the GSTP1 gene can increase the onset of mitochondrial dysfunction and oxidative stress (mechanisms involved in the etiology of ASD). Furthermore, Mn has been severely associated with dopaminergic dysfunction, including structural, functional, and neurochemical alterations of the dopaminergic system, suggesting Mn overexposure is a potential risk factor in ASD pathogenesis [93].

#### 5.3.5. Nickel

Nickel (Ni) constitutes a serious threat to human health [100]. It reaches the biological system through food, water, skin contact, and air, usually during its industrial use, with negative consequences on organs such as kidneys, lungs, liver, and brain.

The presence of Ni in the body can lead to the production of free radicals, causing oxidative stress, antioxidant level imbalance, and indirect alteration of the DNA repair process [100]. Various developmental alterations have been demonstrated in the *C. elegans*, such as alterations of neurotransmitter systems, including cholinergic, dopaminergic, and GABAergic ones, as well as behavioral deficits in old age following Ni exposure in the first years of life [101]. Different studies have focused on the quantitative analysis of Ni concentration in human tissues in ASD patients: in one study [102], Ni level was measured in hair samples of ASD children, finding a notable increase compared with neurotypical controls; another study [78] conducted in the United States showed that children born in areas with high atmospheric Ni content have been diagnosed as autistic. These results therefore suggest that perinatal exposure to Ni is a risk factor for ASD development. However, some research contradicts these findings; for example, one study [103] has measured Ni levels in the hair of ASD patients, finding a slight increase in the hair of neurotypical children compared with ASD ones.

#### 5.3.6. Arsenic

Data show the neurotoxic effect of arsenic on development [104]: in mainly inorganic form, it has been associated with adverse effects on cognitive functions, such as IQ. However, there are still few studies examining this prenatal exposure and correlating it to ADHD or ASD diagnosis; two of these [105,106] did not find associations between prenatal arsenic exposure and ADHD and ASD, respectively. Many ASD studies have measured arsenic in the hair of patients near industrial structures or in drinking water [104]. Most studies have measured exposure to inorganic arsenic species, while for some populations, such as Norwegians, the main source of arsenic is organic forms derived from the consumption of fish and seafood. However, it is mostly the inorganic form of arsenic to be recognized as neurotoxic (Agency for Toxic Substances and Disease Registry, 2007 [107]). The increased risk of ASD and ADHD related to prenatal organic arsenic exposure should be investigated further.

## 6. Postnatal Nutritional Factors

A brief paragraph should also be dedicated to the influence of postnatal nutritional factors on the possible onset of neurodevelopmental disorders.

Case-control studies have shown that dietary patterns can influence the risk of ADHD; in fact, specific dietary interventions have been proposed for this disorder as adjuvant treatments, for example, nutritional supplements, but also biotic drugs and elimination diets [108], facing towards the gut microbiota.

Also, KD could have a therapeutic role in neurodevelopmental diseases due to its ability to influence epigenetic mechanisms and gut microbial composition [2].

Literature has demonstrated that unhealthy eating patterns, especially “Western” ones, rich in processed foods, snacks, and junk food were positively associated with ADHD [109], while healthy dietary patterns, such as the Mediterranean diet and vegetarian diets, rich in fruit, vegetables, and micronutrients, were inversely associated with the risk of ADHD [110].

Alterations in the levels of specific nutrients, such as vitamin D, zinc, iron, and polyunsaturated fatty acids (PUFA), were associated with aggravation or progression of ADHD, so these nutrients have recently been proposed as adjuvants in treatment of the pathology [111] (in addition to methylphenidate), but, as regards the dietary supplements, only vitamin D or vitamin D combined with magnesium appear to improve ADHD symptoms (in case of insufficient/deficient vitamin D levels) [108]. As regards biotics, there is evidence only for the Lactobacillus rhamnosus GG and for multi-species probiotic integration [108]. The diets of elimination have little evidence and lead to nutritional deficiencies, so caution is advised [108]. Overall, more robust scientific evidence is needed to implement these dietary interventions as part of ADHD therapy.

Regarding omega-3, in the third trimester of pregnancy, the DHA begins to accumulate in the fetal brain, but this accumulation continues throughout the postnatal period thanks to breastfeeding [112]. DHA present in breast milk appears fundamental for the correct development of language, cognitive functions, and social behavior; therefore, for better psychosocial health [113]. In fact, some studies suggest breastfeeding for at least six months and, in cases of shorter duration, the maternal consumption of fish at least twice a week [114].

Another study analyzed the role of omega-3 in ADHD, autism, and early psychosis, and the results seem to demonstrate the benefit of omega-3 supplementation [115], especially in psychotic patients, in which they could prevent the transition to the psychotic phase and improve severe symptoms and overall functioning.

A 2017 study [110] investigated the possible association between the Mediterranean diet and ADHD in children and adolescents; the results showed a positive association between low adherence to the Mediterranean diet and diagnosis of ADHD. Even a lower consumption of fruit, vegetables, pasta, and rice, or habits such as skipping breakfast and consuming fast food, resulted in a higher prevalence of ADHD, as well as high consumption of sugars, sweets, carbonated drinks, caffeine, and low consumption of fish and protein. This interesting study analyzed the role of the entire diet on ADHD rather than the role of specific nutrients.

## 7. Conclusions

Maternal nutrition is a modifiable factor, playing a key role during fetal neurological development. Recently, interest as regards the impact of maternal nutritional state on developmental disorders in offspring is continually increasing, with studies that aim to clarify and define maternal nutritional indications.

Regarding some nutrients, the results are clearer, for example about the inverse association between maternal intake of folic acid or multivitamins and the risk of ASD in children (albeit with heterogeneity between studies); while regarding other dietary factors and their correlation with neurodevelopmental disorders, studies have been inconclusive and worthy of further analysis.

Actually, we know these nutritional recommendations during pregnancy:

- An increased protein requirement (different in the three trimesters of pregnancy);
- A carbohydrate intake of at least 175 g/day, reducing monosaccharides and disaccharides (<10% of total carbohydrate);
- An increased DHA requirement of 100–200 mg/day;
- Supplementation of folic acid (400 μg/day) from 30 days before conception;
- Iodine requirement of 200–250 μg/die.

As regard heavy metals, critical values should be better defined in order to implement the preventive strategies to reduce environmental exposure.

Future hopes, through subsequent studies, are to define and quantify precisely the correct maternal intake of nutrients, both from the diet and from supplementation, possibly identifying objective biomarkers reflecting intake and metabolism of the various necessary nutrients.

Furthermore, the study of genetic variants related to nutrient metabolism could allow a better understanding of gene–diet interactions and the related sensitive exposure window in order to establish a precise preventive nutritional intervention concerning neurodevelopmental disorders. Whole genome sequencing (WGS) and maternal/fetal-targeted precision medicine programs catered to individual genomic profiles could lead to personalized interventions, helping to reduce the risk of neurodevelopmental disorders (but also other diseases) in offspring.

So, controlling nutritional factors should improve mothers’ nutritional state to significantly reduce the risk of neurodevelopmental disorders in offspring.

## Figures and Tables

**Table 1:** Alterations and functions of the main nutrients during prenatal phase, based on literature [45,50].

Nutrients	Alterations	Functions
PUFAs	- Synaptogenesis - Myelination	- Component of neuronal membrane - Component of myelin sheet
Proteins	- Alterations of hippocampal neurogenesis - Reduction of brain and neuronal volume - Reduction of maternal lipid availability and fetal brain fatty acid levels (possible alteration of myelination)	- Growth of fetal tissues - Alternative source of energy - Motor development
Folate	- Neurogenesis - Neural migration - Apoptosis - Synaptogenesis - Myelination	- DNA methylation - Neural progenitor cell proliferation
Iron	- Synaptogenesis - Myelination	- Cell metabolism - Hippocampus development
Iodine	- Neurogenesis - Neural migration - Synaptogenesis - Myelination - Endemic cretinism	- Synthesis of thyroid hormones
Zinc	- Decreased mass in the cerebellum, limbic system, and cerebral cortex, with a reduction in cell number	- Metabolism of carbohydrates and proteins - Synthesis of nucleic acids - Cellular division and differentiation - Regulatory of the immune system
Vitamin D	- Neuronal differentiation - Neurotransmission - Alteration of calcium homeostasis	- Regulation of gene expression
Vitamin B12	- Myelination - Neural tube defects	- Enzyme and a cofactor - Metabolism of fats and proteins - Hemoglobin synthesis - Contribution to DNA methylation
Vitamin B6	- Development and maintenance of CNS and immune system	- Metabolism of amino acids, carbohydrates and fats - DNA methylation
Choline	- Neurogenesis and angiogenesis in the fetal hippocampus	- Synthesis of phospholipids and neurotransmitters - DNA methylation

PUFAs: Polyunsaturated fatty acids.

**Table 2:** Main pathogenetic mechanisms and alterations induced by heavy metals during prenatal phase, based on literature [42,67].

Heavy Metals	Alterations	Pathogenetic Mechanisms
Lead	- Behavioral alterations	- Dopaminergic disfunction - Ossidative stress
Mercury	- BBB-cross-over and accumulation	- GST disfunction
Cadmium	- Behavioral alterations	- Alterations of proliferation, differentiation, and apoptosis
Manganese	- Neurodegenerative disease - Cognitive impairment, short term memory loss and attention deficits	- Dopaminergic disfunction - Ossidative stress
Nichel	- Behavioral alterations	- Ossidative stress - Alteration of DNA repair processes - Neurotransmitters disfunction
Arsenic	- Alterations of cognitive functions	- Neurotoxicity - Alteration of cell proliferation

## References

[1] Franzago M., Santurbano D., Vitacolonna E., Stuppia L. (2020). Genes and Diet in the Prevention of Chronic Diseases in Future Generations. Int. J. Mol. Sci..

[2] Santangelo A., Corsello A., Spolidoro G.C.I., Trovato C.M., Agostoni C., Orsini A., Milani G.P., Peroni D.G. (2023). The Influence of Ketogenic Diet on Gut Microbiota: Potential Benefits, Risks and Indications. Nutrients.

[3] Qi X., Yun C., Pang Y., Qiao J. (2021). The Impact of the Gut Microbiota on the Reproductive and Metabolic Endocrine System. Gut Microbes.

[4] Strobel K.M., Juul S.E., Hendrixson D.T. (2023). Maternal Nutritional Status and the Microbiome across the Pregnancy and the Post-Partum Period. Microorganisms.

[5] Cömert T.K., Akpinar F., Erkaya S., Durmaz B., Durmaz R. (2022). The Effect of Pre-Pregnancy Obesity on Gut and Meconium Microbiome and Relationship with Fetal Growth. J. Matern. Fetal Neonatal Med..

[6] Liu T., Jia F., Differding M.K., Zhao N., Doyon M., Bouchard L., Perron P., Guérin R., Massé E., Hivert M.F. (2023). Pre-Pregnancy Body Mass Index and Gut Microbiota of Mothers and Children 5 Years Postpartum. Int. J. Obes..

[7] American Psychiatric Association (APA) (2013). Diagnostic and Statistical Manual of Mental Disorders, (DSM-5).

[8] American Psychiatric Association (APA) (1994). Diagnostic and Statistical Manual of Mental Disorders, (DSM-IV).

[9] Hu V.W., Devlin C.A., Debski J.J. (2019). ASD Phenotype-Genotype Associations in Concordant and Discordant Monozygotic and Dizygotic Twins Stratified by Severity of Autistic Traits. Int. J. Mol. Sci..

[10] Boivin M.J., Kakooza A.M., Warf B.C., Davidson L.L., Grigorenko E.L. (2015). Reducing Neurodevelopmental Disorders and Disability through Research and Interventions. Nature.

[11] Grayson D.R., Guidotti A. (2016). Merging Data from Genetic and Epigenetic Approaches to Better Understand Autistic Spectrum Disorder. Epigenomics.

[12] Masini E., Loi E., Vega-Benedetti A.F., Carta M., Doneddu G., Fadda R., Zavattari P. (2020). An Overview of the Main Genetic, Epigenetic and Environmental Factors Involved in Autism Spectrum Disorder Focusing on Synaptic Activity. Int. J. Mol. Sci..

[13] Kaizuka T., Takumi T. (2018). Postsynaptic Density Proteins and Their Involvement in Neurodevelopmental Disorders. J. Biochem..

[14] Vissers L.E.L.M., Gilissen C., Veltman J.A. (2016). Genetic Studies in Intellectual Disability and Related Disorders. Nat. Rev. Genet..

[15] Hamza M., Halayem S., Mrad R., Bourgou S., Charfi F., Belhadj A. (2017). Epigenetics’ Implication in Autism Spectrum Disorders: A Review. Encephale.

[16] Janecka M., Mill J., Basson M.A., Goriely A., Spiers H., Reichenberg A., Schalkwyk L., Fernandes C. (2017). Advanced Paternal Age Effects in Neurodevelopmental Disorders-Review of Potential Underlying Mechanisms. Transl. Psychiatry.

[17] Gardener H., Spiegelman D., Buka S.L. (2011). Perinatal and Neonatal Risk Factors for Autism: A Comprehensive Meta-Analysis. Pediatrics.

[18] O’Callaghan A., van Sinderen D. (2016). Bifidobacteria and Their Role as Members of the Human Gut Microbiota. Front. Microbiol..

[19] Sudo N., Chida Y., Aiba Y., Sonoda J., Oyama N., Yu X.N., Kubo C., Koga Y. (2004). Postnatal Microbial Colonization Programs the Hypothalamic-Pituitary-Adrenal System for Stress Response in Mice. J. Physiol..

[20] Zhang Y., Hodgson N.W., Trivedi M.S., Abdolmaleky H.M., Fournier M., Cuenod M., Do K.Q., Deth R.C. (2016). Decreased Brain Levels of Vitamin B12 in Aging, Autism and Schizophrenia. PLoS ONE.

[21] Beghetti I., Barone M., Brigidi P., Sansavini A., Corvaglia L., Aceti A., Turroni S. (2023). Early-Life Gut Microbiota and Neurodevelopment in Preterm Infants: A Narrative Review. Front. Nutr..

[22] Rabe H., Lundell A.C., Sjöberg F., Ljung A., Strömbeck A., Gio-Batta M., Maglio C., Nordström I., Andersson K., Nookaew I. (2020). Neonatal Gut Colonization by Bifidobacterium Is Associated with Higher Childhood Cytokine Responses. Gut Microbes.

[23] Dash S., Syed Y.A., Khan M.R. (2022). Understanding the Role of the Gut Microbiome in Brain Development and Its Association with Neurodevelopmental Psychiatric Disorders. Front. Cell Dev. Biol..

[24] Foiadelli T., Santangelo A., Costagliola G., Costa E., Scacciati M., Riva A., Volpedo G., Smaldone M., Bonuccelli A., Clemente A.M. (2023). Neuroinflammation and Status Epilepticus: A Narrative Review Unraveling a Complex Interplay. Front. Pediatr..

[25] Werling D.M. (2016). The Role of Sex-Differential Biology in Risk for Autism Spectrum Disorder. Biol. Sex Differ..

[26] Baron-Cohen S., Auyeung B., Nørgaard-Pedersen B., Hougaard D.M., Abdallah M.W., Melgaard L., Cohen A.S., Chakrabarti B., Ruta L., Lombardo M.V. (2015). Elevated Fetal Steroidogenic Activity in Autism. Mol. Psychiatry.

[27] Tang S., Wang Y., Gong X., Wang G. (2015). A Meta-Analysis of Maternal Smoking during Pregnancy and Autism Spectrum Disorder Risk in Offspring. Int. J. Environ. Res. Public Health.

[28] Gallagher C., McCarthy F.P., Ryan R.M., Khashan A.S. (2018). Maternal Alcohol Consumption During Pregnancy and the Risk of Autism Spectrum Disorders in Offspring: A Retrospective Analysis of the Millennium Cohort Study. J. Autism Dev. Disord..

[29] Meador K.J. (2008). Effects of in Utero Antiepileptic Drug Exposure. Epilepsy Curr..

[30] Veroniki A.A., Cogo E., Rios P., Straus S.E., Finkelstein Y., Kealey R., Reynen E., Soobiah C., Thavorn K., Hutton B. (2017). Comparative Safety of Anti-Epileptic Drugs during Pregnancy: A Systematic Review and Network Meta-Analysis of Congenital Malformations and Prenatal Outcomes. BMC Med..

[31] Xu G., Jing J., Bowers K., Liu B., Bao W. (2014). Maternal Diabetes and the Risk of Autism Spectrum Disorders in the Offspring: A Systematic Review and Meta-Analysis. J. Autism Dev. Disord..

[32] Jiang H.Y., Xu L.L., Shao L., Xia R.M., Yu Z.H., Ling Z.X., Yang F., Deng M., Ruan B. (2016). Maternal Infection during Pregnancy and Risk of Autism Spectrum Disorders: A Systematic Review and Meta-Analysis. Brain Behav. Immun..

[33] Wu S., Ding Y., Wu F., Li R., Xie G., Hou J., Mao P. (2015). Family History of Autoimmune Diseases Is Associated with an Increased Risk of Autism in Children: A Systematic Review and Meta-Analysis. Neurosci. Biobehav. Rev..

[34] Li M., Fallin M.D., Riley A., Landa R., Walker S.O., Silverstein M., Caruso D., Pearson C., Kiang S., Dahm J.L. (2016). The Association of Maternal Obesity and Diabetes with Autism and Other Developmental Disabilities. Pediatrics.

[35] Maldonado-Ruiz R., Garza-Ocañas L., Camacho A. (2019). Inflammatory Domains Modulate Autism Spectrum Disorder Susceptibility during Maternal Nutritional Programming. Neurochem. Int..

[36] Schafer D.P., Stevens B. (2015). Microglia Function in Central Nervous System Development and Plasticity. Cold Spring Harb. Perspect. Biol..

[37] Estes M.L., McAllister A.K. (2016). Maternal Immune Activation: Implications for Neuropsychiatric Disorders. Science.

[38] Vohr B.R., Davis E.P., Wanke C.A., Krebs N.F. (2017). Neurodevelopment: The Impact of Nutrition and Inflammation During Preconception and Pregnancy in Low-Resource Settings. Pediatrics.

[39] Napoli E., Hung C., Wong S., Giulivi C. (2013). Toxicity of the Flame-Retardant BDE-49 on Brain Mitochondria and Neuronal Progenitor Striatal Cells Enhanced by a PTEN-Deficient Background. Toxicol. Sci..

[40] Yoshimasu K., Kiyohara C., Takemura S., Nakai K. (2014). A Meta-Analysis of the Evidence on the Impact of Prenatal and Early Infancy Exposures to Mercury on Autism and Attention Deficit/Hyperactivity Disorder in the Childhood. Neurotoxicology.

[41] Shelton J.F., Geraghty E.M., Tancredi D.J., Delwiche L.D., Schmidt R.J., Ritz B., Hansen R.L., Hertz-Picciotto I. (2014). Neurodevelopmental Disorders and Prenatal Residential Proximity to Agricultural Pesticides: The CHARGE Study. Environ. Health Perspect..

[42] Ijomone O.M., Olung N.F., Akingbade G.T., Okoh C.O.A., Aschner M. (2020). Environmental Influence on Neurodevelopmental Disorders: Potential Association of Heavy Metal Exposure and Autism. J. Trace Elem. Med. Biol..

[43] MRC Vitamin Study Research Group (1991). Prevention of Neural Tube Defects: Results of the Medical Research Council Vitamin Study. Lancet.

[44] Morgan E.H. (1961). Plasma-Iron and Haemoglobin Levels in Pregnancy. The Effect of Oral Iron. Lancet.

[45] Li M., Francis E., Hinkle S.N., Ajjarapu A.S., Zhang C. (2019). Preconception and Prenatal Nutrition and Neurodevelopmental Disorders: A Systematic Review and Meta-Analysis. Nutrients.

[46] Bekdash R.A. (2024). Epigenetics, Nutrition, and the Brain: Improving Mental Health through Diet. Int. J. Mol. Sci..

[47] Gabbianelli R., Damiani E. (2018). Epigenetics and Neurodegeneration: Role of Early-Life Nutrition. J. Nutr. Biochem..

[48] King J.C. (2016). A Summary of Pathways or Mechanisms Linking Preconception Maternal Nutrition with Birth Outcomes. J. Nutr..

[49] Stephenson J., Heslehurst N., Hall J., Schoenaker D.A.J.M., Hutchinson J., Cade J.E., Poston L., Barrett G., Crozier S.R., Barker M. (2018). Before the Beginning: Nutrition and Lifestyle in the Preconception Period and Its Importance for Future Health. Lancet.

[50] Cortés-Albornoz M.C., García-Guáqueta D.P., Velez-Van-meerbeke A., Talero-Gutiérrez C. (2021). Maternal Nutrition and Neurodevelopment: A Scoping Review. Nutrients.

[51] Lane M., Robker R.L., Robertson S.A. (2014). Parenting from before Conception. Science.

[52] Debnath M., Venkatasubramanian G., Berk M. (2015). Fetal Programming of Schizophrenia: Select Mechanisms. Neurosci. Biobehav. Rev..

[53] Monk C., Georgieff M.K., Osterholm E.A. (2013). Research Review: Maternal Prenatal Distress and Poor Nutrition—Mutually Influencing Risk Factors Affecting Infant Neurocognitive Development. J. Child. Psychol. Psychiatry.

[54] Sullivan E.L., Nousen E.K., Chamlou K.A. (2014). Maternal High Fat Diet Consumption during the Perinatal Period Programs Offspring Behavior. Physiol. Behav..

[55] Godfrey K.M., Reynolds R.M., Prescott S.L., Nyirenda M., Jaddoe V.W.V., Eriksson J.G., Broekman B.F.P. (2017). Influence of Maternal Obesity on the Long-Term Health of Offspring. Lancet Diabetes Endocrinol..

[56] Sanchez C.E., Barry C., Sabhlok A., Russell K., Majors A., Kollins S.H., Fuemmeler B.F. (2018). Maternal Pre-Pregnancy Obesity and Child Neurodevelopmental Outcomes: A Meta-Analysis. Obes. Rev..

[57] Hannou S.A., Haslam D.E., McKeown N.M., Herman M.A. (2018). Fructose Metabolism and Metabolic Disease. J. Clin. Investig..

[58] DeCapo M., Thompson J.R., Dunn G., Sullivan E.L. (2019). Perinatal Nutrition and Programmed Risk for Neuropsychiatric Disorders: A Focus on Animal Models. Biol. Psychiatry.

[59] Martins B.P., Bandarra N.M., Figueiredo-Braga M. (2020). The Role of Marine Omega-3 in Human Neurodevelopment, Including Autism Spectrum Disorders and Attention-Deficit/Hyperactivity Disorder—A Review. Crit. Rev. Food Sci. Nutr..

[60] van Elst K., Bruining H., Birtoli B., Terreaux C., Buitelaar J.K., Kas M.J. (2014). Food for Thought: Dietary Changes in Essential Fatty Acid Ratios and the Increase in Autism Spectrum Disorders. Neurosci. Biobehav. Rev..

[61] Kim H.Y., Spector A.A., Xiong Z.M. (2011). A Synaptogenic Amide N-Docosahexaenoylethanolamide Promotes Hippocampal Development. Prostaglandins Other Lipid Mediat..

[62] Prado E.L., Dewey K.G. (2014). Nutrition and Brain Development in Early Life. Nutr. Rev..

[63] Berti C., Biesalski H.K., Gärtner R., Lapillonne A., Pietrzik K., Poston L., Redman C., Koletzko B., Cetin I. (2011). Micronutrients in Pregnancy: Current Knowledge and Unresolved Questions. Clin. Nutr..

[64] Rodriguez-Diaz E., Pearce E.N. (2020). Iodine Status and Supplementation before, during, and after Pregnancy. Best. Pract. Res. Clin. Endocrinol. Metab..

[65] Uriu-Adams J.Y., Keen C.L. (2005). Copper, Oxidative Stress, and Human Health. Mol. Aspects Med..

[66] Zoroddu M.A., Aaseth J., Crisponi G., Medici S., Peana M., Nurchi V.M. (2019). The Essential Metals for Humans: A Brief Overview. J. Inorg. Biochem..

[67] Skogheim T.S., Weyde K.V.F., Engel S.M., Aase H., Surén P., Øie M.G., Biele G., Reichborn-Kjennerud T., Caspersen I.H., Hornig M. (2021). Metal and Essential Element Concentrations during Pregnancy and Associations with Autism Spectrum Disorder and Attention-Deficit/Hyperactivity Disorder in Children. Environ. Int..

[68] Li Y., Cha C., Lv X.J., Liu J., He J., Pang Q., Meng L., Kuang H., Fan R. (2020). Association between 10 Urinary Heavy Metal Exposure and Attention Deficit Hyperactivity Disorder for Children. Environ. Sci. Pollut. Res. Int..

[69] Richard K., Holland O., Landers K., Vanderlelie J.J., Hofstee P., Cuffe J.S.M., Perkins A.V. (2017). Review: Effects of Maternal Micronutrient Supplementation on Placental Function. Placenta.

[70] Georgieff M.K., Ramel S.E., Cusick S.E. (2018). Nutritional Influences on Brain Development. Acta Paediatr..

[71] Sun G.X., Wang B.H., Zhang Y.F. (2015). Relationship between Serum Zinc Levels and Attention Deficit Hyperactivity Disorder in Children. Zhongguo Dang Dai Er Ke Za Zhi.

[72] Tous M., Villalobos M., Iglesias L., Fernández-Barrés S., Arija V. (2020). Vitamin D Status during Pregnancy and Offspring Outcomes: A Systematic Review and Meta-Analysis of Observational Studies. Eur. J. Clin. Nutr..

[73] Ryan J.M., Rice G.E., Mitchell M.D. (2013). The Role of Gangliosides in Brain Development and the Potential Benefits of Perinatal Supplementation. Nutr. Res..

[74] Qian J., Chen Q., Ward S.M., Duan E., Zhang Y. (2020). Impacts of Caffeine during Pregnancy. Trends Endocrinol. Metab..

[75] Wikoff D., Welsh B.T., Henderson R., Brorby G.P., Britt J., Myers E., Goldberger J., Lieberman H.R., O’Brien C., Peck J. (2017). Systematic Review of the Potential Adverse Effects of Caffeine Consumption in Healthy Adults, Pregnant Women, Adolescents, and Children. Food Chem. Toxicol..

[76] Linnet K.M., Wisborg K., Secher N.J., Hove Thomsen P., Obel C., Dalsgaard S., Henriksen T.B. (2009). Coffee Consumption during Pregnancy and the Risk of Hyperkinetic Disorder and ADHD: A Prospective Cohort Study. Acta Paediatr..

[77] Galéra C., Bernard J.Y., van der Waerden J., Bouvard M.P., Lioret S., Forhan A., De Agostini M., Melchior M., Heude B. (2016). Prenatal Caffeine Exposure and Child IQ at Age 5.5 Years: The EDEN Mother-Child Cohort. Biol. Psychiatry.

[78] Roberts A.L., Lyall K., Hart J.E., Laden F., Just A.C., Bobb J.F., Koenen K.C., Ascherio A., Weisskopf M.G. (2013). Perinatal Air Pollutant Exposures and Autism Spectrum Disorder in the Children of Nurses’ Health Study II Participants. Environ. Health Perspect..

[79] El-Ansary A., Bjørklund G., Tinkov A.A., Skalny A.V., Al Dera H. (2017). Relationship between Selenium, Lead, and Mercury in Red Blood Cells of Saudi Autistic Children. Metab. Brain Dis..

[80] Nair A.R., Lee W.K., Smeets K., Swennen Q., Sanchez A., Thévenod F., Cuypers A. (2015). Glutathione and Mitochondria Determine Acute Defense Responses and Adaptive Processes in Cadmium-Induced Oxidative Stress and Toxicity of the Kidney. Arch. Toxicol..

[81] Akinyemi A.J., Miah M.R., Ijomone O.M., Tsatsakis A., Soares F.A.A., Tinkov A.A., Skalny A.V., Venkataramani V., Aschner M. (2019). Lead (Pb) Exposure Induces Dopaminergic Neurotoxicity in Caenorhabditis Elegans: Involvement of the Dopamine Transporter. Toxicol. Rep..

[82] Kim K.N., Kwon H.J., Hong Y.C. (2016). Low-Level Lead Exposure and Autistic Behaviors in School-Age Children. Neurotoxicology.

[83] Fuentes-Albero M., Puig-Alcaraz C., Cauli O. (2015). Lead Excretion in Spanish Children with Autism Spectrum Disorder. Brain Sci..

[84] Qin Y.Y., Jian B., Wu C., Jiang C.Z., Kang Y., Zhou J.X., Yang F., Liang Y. (2018). A Comparison of Blood Metal Levels in Autism Spectrum Disorder and Unaffected Children in Shenzhen of China and Factors Involved in Bioaccumulation of Metals. Environ. Sci. Pollut. Res. Int..

[85] Rice K.M., Walker E.M., Wu M., Gillette C., Blough E.R. (2014). Environmental Mercury and Its Toxic Effects. J. Prev. Med. Public Health.

[86] Jafari T., Rostampour N., Fallah A.A., Hesami A. (2017). The Association between Mercury Levels and Autism Spectrum Disorders: A Systematic Review and Meta-Analysis. J. Trace Elem. Med. Biol..

[87] Golding J., Rai D., Gregory S., Ellis G., Emond A., Iles-Caven Y., Hibbeln J., Taylor C. (2018). Prenatal Mercury Exposure and Features of Autism: A Prospective Population Study. Mol. Autism.

[88] Geier D.A., Kern J.K., King P.G., Sykes L.K., Geier M.R. (2012). Hair Toxic Metal Concentrations and Autism Spectrum Disorder Severity in Young Children. Int. J. Environ. Res. Public Health.

[89] Kern J.K., Geier D.A., Audhya T., King P.G., Sykes L.K., Geier M.R. (2012). Evidence of Parallels between Mercury Intoxication and the Brain Pathology in Autism. Acta Neurobiol. Exp..

[90] Kern J.K., Grannemann B.D., Trivedi M.H., Adams J.B. (2007). Sulfhydryl-Reactive Metals in Autism. J. Toxicol. Environ. Health A.

[91] Yorbik Ö., Kurt I., Haşimi A., Öztürk Ö. (2010). Chromium, Cadmium, and Lead Levels in Urine of Children with Autism and Typically Developing Controls. Biol. Trace Elem. Res..

[92] Yasuda H., Kobayashi M., Yasuda Y., Tsutsui T. (2013). Estimation of Autistic Children by Metallomics Analysis. Sci. Rep..

[93] Ijomone O.M., Aluko O.M., Okoh C.O.A., Martins A.C., Aschner M. (2019). Role for Calcium Signaling in Manganese Neurotoxicity. J. Trace Elem. Med. Biol..

[94] Martins A.C., Morcillo P., Ijomone O.M., Venkataramani V., Harrison F.E., Lee E., Bowman A.B., Aschner M. (2019). New Insights on the Role of Manganese in Alzheimer’s Disease and Parkinson’s Disease. Int. J. Environ. Res. Public Health.

[95] Zota A.R., Riederer A.M., Ettinger A.S., Schaider L.A., Shine J.P., Amarasiriwardena C.J., Wright R.O., Spengler J.D. (2016). Associations between Metals in Residential Environmental Media and Exposure Biomarkers over Time in Infants Living near a Mining-Impacted Site. J. Expo. Sci. Environ. Epidemiol..

[96] Henn B.C., Ettinger A.S., Schwartz J., Téllez-Rojo M.M., Lamadrid-Figueroa H., Hernández-Avila M., Schnaas L., Amarasiriwardena C., Bellinger D.C., Hu H. (2010). Early Postnatal Blood Manganese Levels and Children’s Neurodevelopment. Epidemiology.

[97] Arora M., Reichenberg A., Willfors C., Austin C., Gennings C., Berggren S., Lichtenstein P., Anckarsäter H., Tammimies K., Bölte S. (2017). Fetal and Postnatal Metal Dysregulation in Autism. Nat. Commun..

[98] Abdullah M.M., Ly A.R., Goldberg W.A., Clarke-Stewart K.A., Dudgeon J.V., Mull C.G., Chan T.J., Kent E.E., Mason A.Z., Ericson J.E. (2012). Heavy Metal in Children’s Tooth Enamel: Related to Autism and Disruptive Behaviors?. J. Autism Dev. Disord..

[99] Rahbar M.H., Samms-Vaughan M., Ma J., Bressler J., Dickerson A.S., Hessabi M., Loveland K.A., Grove M.L., Shakespeare-Pellington S., Beecher C. (2015). Synergic Effect of GSTP1 and Blood Manganese Concentrations in Autism Spectrum Disorder. Res. Autism Spectr. Disord..

[100] Ijomone O.M., Okori S.O., Ijomone O.K., Ebokaiwe A.P. (2018). Sub-Acute Nickel Exposure Impairs Behavior, Alters Neuronal Microarchitecture, and Induces Oxidative Stress in Rats’ Brain. Drug Chem. Toxicol..

[101] Ijomone O.M., Miah M.R., Akingbade G.T., Bucinca H., Aschner M. (2020). Nickel-Induced Developmental Neurotoxicity in *C. elegans* Includes Cholinergic, Dopaminergic and GABAergic Degeneration, Altered Behaviour, and Increased SKN-1 Activity. Neurotox. Res..

[102] Al-Farsi Y.M., Waly M.I., Al-Sharbati M.M., Al-Shafaee M.A., Al-Farsi O.A., Al-Khaduri M.M., Gupta I., Ouhtit A., Al-Adawi S., Al-Said M.F. (2013). Levels of Heavy Metals and Essential Minerals in Hair Samples of Children with Autism in Oman: A Case-Control Study. Biol. Trace Elem. Res..

[103] Skalny A.V., Simashkova N.V., Klyushnik T.P., Grabeklis A.R., Radysh I.V., Skalnaya M.G., Tinkov A.A. (2017). Analysis of Hair Trace Elements in Children with Autism Spectrum Disorders and Communication Disorders. Biol. Trace Elem. Res..

[104] Bjørklund G., Skalny A.V., Rahman M.M., Dadar M., Yassa H.A., Aaseth J., Chirumbolo S., Skalnaya M.G., Tinkov A.A. (2018). Toxic Metal(Loid)-Based Pollutants and Their Possible Role in Autism Spectrum Disorder. Environ. Res..

[105] Forns J., Fort M., Casas M., Cáceres A., Guxens M., Gascon M., Garcia-Esteban R., Julvez J., Grimalt J.O., Sunyer J. (2014). Exposure to Metals during Pregnancy and Neuropsychological Development at the Age of 4 Years. Neurotoxicology.

[106] Long M., Ghisari M., Kjeldsen L., Wielsøe M., Nørgaard-Pedersen B., Mortensen E.L., Abdallah M.W., Bonefeld-Jørgensen E.C. (2019). Autism Spectrum Disorders, Endocrine Disrupting Compounds, and Heavy Metals in Amniotic Fluid: A Case-Control Study. Mol. Autism.

[107] U.S. Department of Health and Human Services Agency for Toxic Substances and Disease Registry (ATSDR) 2007. https://www.atsdr.cdc.gov/spl/previous/07list.html.

[108] Pinto S., Correia-de-Sá T., Sampaio-Maia B., Vasconcelos C., Moreira P., Ferreira-Gomes J. (2022). Eating Patterns and Dietary Interventions in ADHD: A Narrative Review. Nutrients.

[109] Del-Ponte B., Quinte G.C., Cruz S., Grellert M., Santos I.S. (2019). Dietary Patterns and Attention Deficit/Hyperactivity Disorder (ADHD): A Systematic Review and Meta-Analysis. J. Affect. Disord..

[110] Rios-Hernandez A., Alda J.A., Farran-Codina A., Ferreira-Garcia E., Izquierdo-Pulido M. (2017). The Mediterranean Diet and ADHD in Children and Adolescents. Pediatrics.

[111] Klaus W., Lange Y.N.A.R. (2022). Diet and Food in Attention-Deficit Hyperactivity Disorder. J. Future Foods.

[112] Janssen C.I.F., Kiliaan A.J. (2014). Long-Chain Polyunsaturated Fatty Acids (LCPUFA) from Genesis to Senescence: The Influence of LCPUFA on Neural Development, Aging, and Neurodegeneration. Prog. Lipid Res..

[113] Belfort M.B., Rifas-Shiman S.L., Kleinman K.P., Bellinger D.C., Harris M.H., Taveras E.M., Gillman M.W., Oken E. (2016). Infant Breastfeeding Duration and Mid-Childhood Executive Function, Behavior, and Social-Emotional Development. J. Dev. Behav. Pediatr..

[114] Mendez M.A., Torrent M., Julvez J., Ribas-Fitó N., Kogevinas M., Sunyer J. (2009). Maternal Fish and Other Seafood Intakes during Pregnancy and Child Neurodevelopment at Age 4 Years. Public Health Nutr..

[115] Hadders-Algra M. (2011). Prenatal and Early Postnatal Supplementation with Long-Chain Polyunsaturated Fatty Acids: Neurodevelopmental Considerations. Am. J. Clin. Nutr..

